# Safety and Immunogenicity of the Attenuated Yellow Fever Vaccine in Several Neotropical Primate Species

**DOI:** 10.3390/vaccines13050487

**Published:** 2025-04-30

**Authors:** Nayara Ferreira de Paula, André Duarte Vieira, Daniel Oliveira dos Santos, Lucas dos Reis de Souza, Carlyle Mendes Coelho, Herlandes Penha Tinoco, Paula Cristina Senra Lima, Rafael Otávio Cançado Motta, Valéria do Socorro Pereira, Marcelo Pires Nogueira de Carvalho, Camilla Bayma Fernandes, Adriana de Souza Azevedo, Matheus Soares Arruda, Thais Alkifeles Costa, Betania Paiva Drumond, Fabiola de Oliveira Paes Leme, Marcos da Silva Freire, Tatiane Alves da Paixão, Ayisa Rodrigues Oliveira, Renato Lima Santos

**Affiliations:** 1 Departamento de Clínica e Cirurgia Veterinárias, Escola de Veterinária, Universidade Federal de Minas Gerais (UFMG), Av. Antônio Carlos 6627, Belo Horizonte 31270-901, Brazil; nay.ferreiradepaula@gmail.com (N.F.d.P.); aduarte858@gmail.com (A.D.V.); ayisarodrigues@gmail.com (A.R.O.); 2 Fundação de Parques Municipais e Zoobotânica, Jardim Zoológico, Av. Otacílio Negrão de Lima 8000, Belo Horizonte 31365-450, Brazil; carlyle.m@pbh.gov.br (C.M.C.); vpereira@pbh.gov.br (V.d.S.P.); 3 Fundação Oswaldo Cruz, Instituto de Tecnologia em Imunobiológicos (Bio-Manguinhos), Av. Brasil 4365, Rio de Janeiro 21040-900, Brazil; 4 Departamento de Microbiologia, Instituto de Ciências Biológicas, Universidade Federal de Minas Gerais (UFMG), Av. Antônio Carlos 6627, Belo Horizonte 31270-901, Brazil; matheusmtsa095@gmail.com (M.S.A.);; 5 Departamento de Patologia Geral, Instituto de Ciências Biológicas, Universidade Federal de Minas Gerais (UFMG), Av. Antônio Carlos 6627, Belo Horizonte 31270-901, Brazil

**Keywords:** flavivirus, yellow fever virus, yellow fever, 17DD, non-human primates, vaccination, RNAemia, neutralizing antibodies

## Abstract

Background/Objective: Yellow fever (YF) is an acute infectious disease caused by the yellow fever virus which is transmitted by mosquitoes. Neotropical primates are susceptible to infection, which is often presented as epizootic outbreaks. The aim was to evaluate and characterize the immune response against YF in different species of neotropical primates from the Belo Horizonte Zoo. Methods: Vaccine 17DD was administered to 24 neotropical primates, with a single subcutaneous dose. Clinical exams, RNAemia, and detection of IgG and neutralizing antibodies against YFV were performed. In addition, an ethogram was performed to assess clinical changes and animal welfare. Results: At 4 days post-vaccination, RNAemia was detected in nine animals. There was seroconversion and persistence of immune response in *Alouatta guariba clamitans*, *Sapajus xanthosternos*, *Saguinus imperator* and *Aotus infulatus*. However, the vaccine was not immunogenic for *Lagothrix cana*. In *Pithecia irrorata* seroconversion did not persist long term, while the *Ateles* sp. had a transient immune response. No significant clinical manifestations were observed in any of the vaccinated animals. Conclusions: This study demonstrated a safe, immunogenic and persistent immune response induced by the attenuated 17DD vaccine strain in *A. guariba clamitans*, *S. xanthosternos*, *S. imperator*, and *A. infulatus*.

## 1. Introduction

Yellow fever (YF) is a mosquito-borne hemorrhagic disease in humans and non-human primates (NHPs) caused by the yellow fever virus (YFV). It is transmitted to susceptible hosts by the vectors *Haemagogus* sp. or *Sabethes* sp. in forested environments, or *Aedes aegypti* in urban areas [1]. In Brazil, the most recent outbreak occurred primarily in the Southeastern region of Brazil from 2016 to 2018, resulting in more than 1000 human cases, with a case lethality rate of 35.1% [2].

Neotropical primates are susceptible to YFV, so the disease is a significant concern for conservation of those populations, especially in non-endemic regions with low viral circulation [3]. In contrast, African primates are highly resistant to YFV and rarely develop disease [4]. Neotropical primates are divided into five families: Callithrichidae, Cebidae, Aotidae, Pithecidae, and Atelidae. At least 36 species are endemic to Brazil, and all are classified as vulnerable to some degree according to the Convention on International Trade in Endangered Species of Wild Fauna and Flora (CITES) [5].

The most common lesions in YFV-infected NHPs are hepatic changes characterized by severe midzonal to massive necrosis with lipidosis and mild inflammatory infiltrate, including lymphocytes, macrophages, plasma cells and, rarely, neutrophils. Severe necrotizing YFV-induced liver lesions are often observed in *Alouatta* sp. (howler monkeys), while *Callithrix* sp. (marmosets) tend to develop milder liver lesions, which are less frequent than those of *Alouatta* sp. Furthermore, the frequency of YFV infection is described as significantly higher in *Alouatta* sp. compared with *Callithrix* sp. or *Sapajus* sp. (capuchin monkeys) [6,7]. These data support the hypothesis that *Alouatta* spp. are highly susceptible to infection and lesions induced by YFV, while *Callithrix* spp. is susceptible to infection but is more resistant to the development of YFV-elicited lesions [6].

During YF outbreaks, death of neotropical primates usually precedes human cases [8]. Therefore, monitoring the occurrence of YF in NHP serves as an early warning of viral circulation in a particular area, which allows prevention through vaccination of potentially exposed human populations [3].

The attenuated YF vaccine 17DD is safe and provides lifelong immunity to humans. It is produced in specific pathogen-free embryonated chicken eggs, using a seed batch system implemented in the early 1940s that has not undergone significant changes to date. The commercial YF vaccine produced in Brazil uses the live attenuated 17DD strain [9].

A previous study carried out in howler monkeys revealed the development of neutralizing antibody titers against YFV after vaccination with the attenuated 17DD vaccine, with no adverse effects related to the vaccine or reversal of virulence and disease development [10]. Recently, this vaccine has also been demonstrated to be safe for golden-headed lion tamarins (*Leontopithecus chrysomelas*), as were two other experimental inactivated vaccines: a purified whole-virus vaccine inactivated by β-propiolactone and a plant-derived recombinant subunit vaccine. The inactivated vaccine resulted in the production of neutralizing antibodies and prevented post-challenge viremia and RNAemia in five of six animals, while the plant-based vaccine induced neutralizing antibodies in five of six animals and prevented viremia and RNAemia in three of six [11].

In Brazil, between December 2016 and June 2017, the YF outbreak resulted in hundreds of human cases, with lethality rate of over a third of the cases [12], and 1412 confirmed infections in NHPs [13]. The high lethality persisted in 2018 [14], with its epicenter in the State of Minas Gerais [15], covering more than 2000 km^2^ [16]. Entire populations of black howler monkeys in the states of Minas Gerais and Espírito Santo were reduced to a fraction of their original sizes and, in some smaller forest fragments, entire populations became locally extinct [17]. During that period, widespread circulation of YF was demonstrated in the State of Minas Gerais, with the virus being detected in 298 carcasses of NHPs from 49 municipalities, both in urban and rural areas. The areas with the highest number of YFV-positive NHP were the same areas with the highest number of human YF cases from 2016 to 2018 [18]. Therefore, the devastating effects of YFV on neotropical primates support the initiatives of vaccinating neotropical primates under human care or in the wild.

The aim of this study was to evaluate the safety and immunogenicity of the attenuated 17DD vaccine in various species of neotropical primates kept under human care at the Belo Horizonte Zoo (Belo Horizonte, State of Minas Gerais, Brazil), characterizing the immune response induced by the vaccine through the detection of neutralizing antibody titers and the development of post-vaccination IgG antibodies. Reactogenicity parameters were also evaluated, in addition to the evaluation of post-vaccination viral RNA levels in the peripheral blood (RNAemia).

## 2. Materials and Methods

### 2.1. Ethics

All procedures strictly adhered to the humane care of animals and all applicable laws and regulations, as well as the ethical policies of the journal. This study protocol has been approved by the Animal Use Ethics Committee of the Universidade Federal de Minas Gerais (No. 159/2022). This study also had an environmental license provided by the Instituto Chico Mendes de Conservação da Biodiversidade (ICMBio-SISBIO 83903-1/2022), as well as authorization from the Fundação de Parques Municipais e Zoobotânica de Belo Horizonte (FU007/2022).

### 2.2. Animals and Restraint Procedures

All neotropical primates housed at the Belo Horizonte Zoo were included in this study, with the exception of *Leontopithecus* spp., for which we were not granted authorization for vaccination (Table 1). The animals were kept in various enclosures, which consisted of an exhibition area and a maneuvering area, and were provided with a balanced diet, primarily consisting of animal feed, fruits, and vegetables, with water ad libitum.

Each animal was contained in the maneuvering area of each enclosure and captured with a nylon trap of appropriate size for each species. The primates were then manually restrained using a leather scraping glove and/or a procedure glove by the technical team at the zoo. For chemical restraint, each animal was fasted for 12 h. After physical restraint, *Saguinus imperator* (emperor tamarins), *Pithecia irrorata* (gray’s bald-faced saki), and *Aotus infulatus* (owl monkey) were exposed to 3% isoflurane. *Lagothrix cana* (woolly monkeys), *Alouatta guariba clamitans* (brown howler monkeys), *Sapajus xanthosternos* (yellow-breasted capuchins), and *Ateles* sp. (spider monkey) received 10% ketamine and 0.5% midazolam at doses of 5 mg/kg and 0.3 mg/kg, respectively, by intramuscular injection, followed by inhalation anesthesia if necessary.

### 2.3. Experimental Design

**Figure 1:**
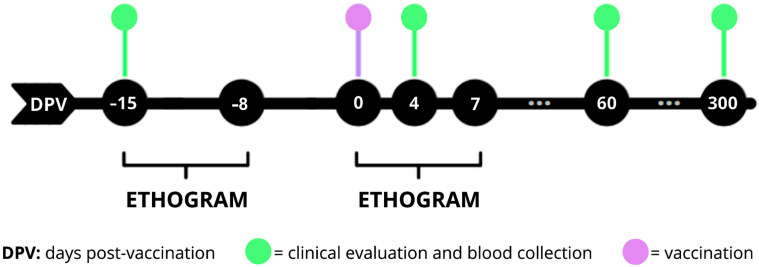
Experimental design. At −15, 4, 60, and 300 days post-vaccination (DPV), blood sampling and clinical examination of each of the animals were performed. Vaccination was performed at day 0 by subcutaneous injection in the hypogastric region. An ethogram was evaluated one week after screening and one week post-vaccination.

Blood samples were obtained by venous puncture of the femoral and/or saphenous veins, with a volume of up to 1% of the body weight of each animal, during the screening, 15 days before vaccination (−15 DPV), and also at 4, 60, and 300 days post-vaccination (DPV). For vaccination, they were physically restrained and, when necessary, chemically restrained (0 DPV) (Figure 1).

Additionally, an observational behavior analysis with the establishment of an ethogram was conducted during 7-day periods, between −15 and −8 DPV and 0 to 7 DPV. Observations were performed for 1 h in the morning and 1 h in the afternoon, preferably after food distribution, always at the same time and by the same observer. Activities of each animal were recorded every minute, as well as climatic conditions, and possible external interferences. At the end of the evaluation, the frequency of time spent on each behavior was calculated, subdividing them into active behaviors (movement on branches and on the ground, and copulation), non-active behaviors (still on the ground, still on the branch and not visible), water intake, feeding, urination, and defecation. During the first 7 DPV, in addition to the parameters included in the ethogram, other parameters were also recorded, including handling of the vaccination site, and clinical signs such as vomiting, prostration, hemorrhagic or neurological manifestations.

Pre-vaccination screening was carried out fifteen days before vaccination (−15 DPV), testing for YFV by reverse transcription followed by polymerase chain reaction (RT–qPCR). Clinical evaluation of each animal included measurement of heart rate, respiratory rate, body temperature, weight, complete blood count, and a serum biochemical profile (urea, creatinine, alanine aminotransferase, aspartate aminotransferase, amylase, gamma glutamyl-transferase, total protein, albumin, globulin, alkaline phosphatase). These tests were performed to exclude any possible pre-existing illness that could affect the vaccination. Animals that had RNAemia, chronic diseases, advanced senility, any debilitating condition, or were under 9 months of age were not vaccinated.

The vaccine was administered subcutaneously (SC) in the hypogastric region with a single dose of the human vaccine containing the live attenuated YFV 17DD strain (Bio-Manguinhos/Fiocruz, Rio de Janeiro, Brazil). The volume applied of the reconstituted vaccine was 100 μL in individuals of larger species (*L. cana*, *A. guariba clamitans*, *S. xanthosternos*, *P. irrorata*, and *Ateles* sp.), and 20 μL for smaller individuals (*S. imperator* and *A. infulatus*). The doses were based on studies previously conducted in howler monkeys [10].

At 4, 60, and 300 DPV, all vaccinated animals were subjected to blood sampling and clinical evaluation to compare with the pre-vaccination evaluation and to assess possible clinical changes due to immunization.

### 2.4. Assessment of RNAemia

RNAemia was quantified by RT–qPCR. Viral RNA was extracted from serum samples at −15, 4, 60, and 300 DPV using QIAamp Viral RNA Mini Kit (Qiagen, Germantown, MD, USA). A negative extraction control (nuclease-free water) was included in each batch of extraction. Each RNA sample, along with negative extraction controls (2.5 µL), was tested in duplicate for YFV using RT–qPCR (GoTaq Probe 1-Step RT–qPCR System kit, Promega Corporation, Madison, WI, USA) with primers and probe targeting the 5′ untranslated region of the YFV genome [19]. Each RT–qPCR run also included a non-template control (nuclease-free water) and a positive control (YFV 17DD RNA). Total Samples with quantification cycles (Ct) below or equal to 37 were considered positive, while those with a Ct greater than 37 or without amplification were considered negative [19].

### 2.5. Detection of Neutralizing Antibody Titers Against Yellow Fever Virus

The levels of neutralizing antibodies against YF were quantified by micro-PRNT50 (micro-Plaque Reduction Neutralization Test) on serum samples collected at −15, 4, 60, and 300 DPV. Briefly, micro-PRNT50 was performed in 96-well plates, where approximately 30 plaque-forming units (PFU) of YFV were added to serially diluted primate serum samples, from 1:5 to 1:640, using a dilution factor of 2. The plates containing the mixtures of serum sample and YFV were then incubated in a CO_2_ incubator for 1 h at 37 °C. After this period, a suspension of Vero CCL-81 cells (ATCC) was added to each well and incubated for 3 h in a CO_2_ incubator. The supernatant was discarded, and semi-solid medium (199 medium supplemented with 5% fetal bovine serum and 2.5% carboxymethylcellulose) was added. The plates were incubated for six days in a CO_2_ incubator at 37 °C and then fixed with a 5% formaldehyde solution and stained with crystal violet. Plates were photographed using BioSpot (CTL-Cellular Technology Limited, Shaker Heights, OH, USA) and the PFU were quantified. The neutralizing antibody titer against YF was defined as the dilution of serum that reduced the number of viral plaques by 50% compared to the control viral plaque count. Titers above 1:5 were considered positive for the presence of neutralizing antibodies against YF [10].

### 2.6. Detection of IgG Against Yellow Fever Virus

Anti-YF IgG was measured by ELISA in sera from all animals obtained at −15, 4, 60, and 300 DPV. The assays were performed using 96-well plates coated with 2.5 μg/mL, 50 μL/well of YFV in coating buffer (carbonate-bicarbonate buffer pH 9.6) and incubated overnight at 4 °C. The excess antibody was then removed. During all washing steps, the microplates were mechanically rinsed 5 times with washing buffer (PBS pH 7.4 with 0.05% Tween-20—PBS/T, 300 μL/well). Plates were blocked with blocking/diluent solution (SBD) 100 μL/well (PBS/T, 0.05% BSA (bovine serum albumin), 3% FBS, and 5% skimmed milk) for 1 h at 37 °C. The samples were diluted 1:20 to perform 4 serial two-fold dilutions and, for the standard curve, 8 serial two-fold dilutions of the anti-YF serum (YF—NIBSC, The National Institute for Biological Standards and Control, Hertfordshire, UK), ranging from 1 to 0.015 mIU/mL, were prepared in SBD. After 1 h at room temperature, the plate was washed and incubated with 100 μL/well of IgG Anti-Monkey antibody conjugated with horseradish peroxidase (A2054-Sigma-Aldrich, Saint Louis, MO, USA), diluted 1:5000 in SBD, and incubated for 1 h at room temperature. After washing, 100 μL/well of substrate solution (TMB plus from Kementec Solutions/Bio-Connect Diagnosis BV, Taastrup, Denmark) was added, followed by 100 μL/well of stop solution (2 M H_2_SO_4_) after 15 min. Final point measurements were made at 450 nm. The absorbance of the sample dilutions was plotted on the standard curve. Antibody titers were calculated using SoftMax Pro 7.1 software by logistic regression for 4 parameters, expressed in IU/mL in relation to the reference anti-serum [10].

### 2.7. Statistical Analysis

GraphPad Prism software (version 8.0.1) was used to analyze data. The ethogram results were compared by analysis of variance, comparing active behaviors, non-active behaviors, water intake, feeding, urination, and defecation, before and after vaccination, with Sidak’s multiple pairwise comparison test. The results of detection of neutralizing antibody titers against YFV and hematological and biochemical parameters of species with more than one individual were compared using the Friedman Test. Additionally, total antibodies against YFV were compared between time points by the Friedman test. Serologic data were compared between animals with or without RNAemia using the Mann–Whitney test, and frequencies of serologically positive animals (ELISA or serum neutralization) were compared using the Fisher’s exact test. Pearson correlation was performed between Ct values of animals with RNAemia and OD values (indicator of IgG levels) or neutralizing antibody titers.

## 3. Results

### 3.1. Ethogram

No significant differences were found in the frequency of any of the parameters analyzed before and after vaccination (*p* > 0.05).

**Figure 2:**
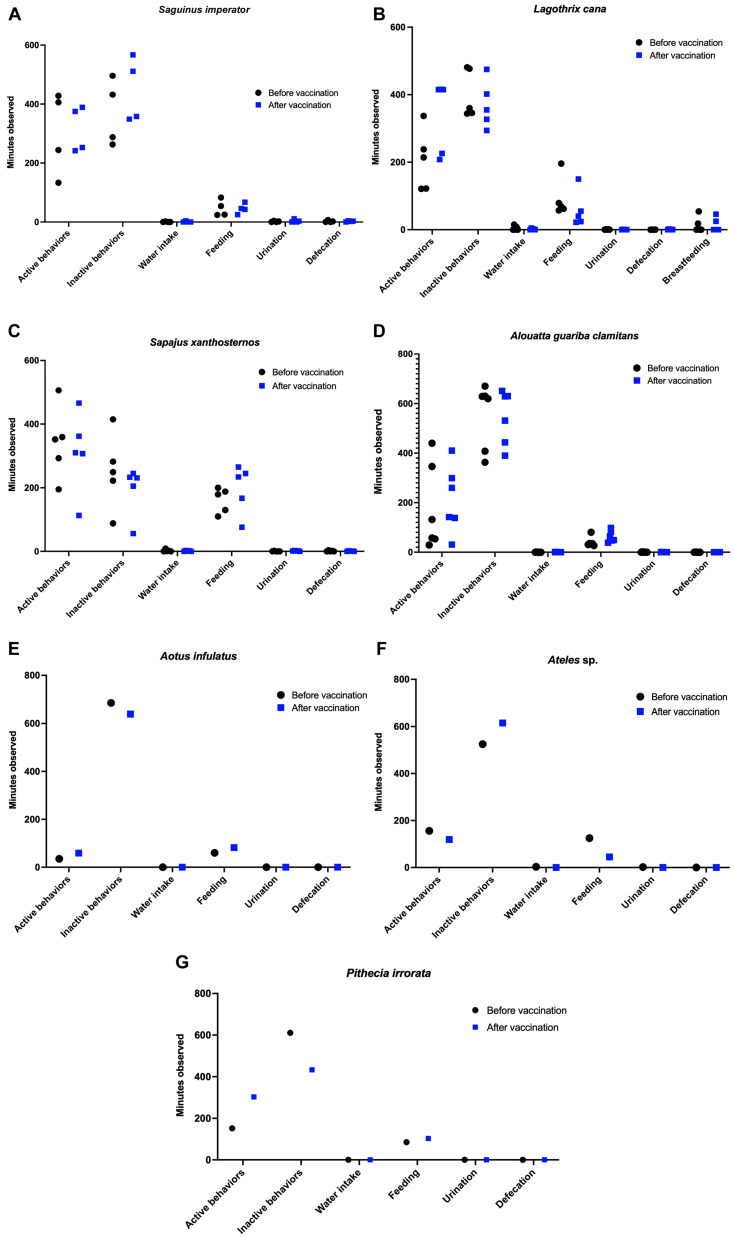
Ethogram of neotropical primates before and after vaccination with the attenuated YF 17DD strain. Frequency of behaviors observed 7 days before vaccination and 7 days after vaccination. (**A**) *Saguinus imperator*; (**B**) *Lagothrix cana*; (**C**) *Sapajus xanthosternos*; (**D**) *Alouatta guariba clamitans*; (**E**) *Aotus infulatus*; (**F**) *Ateles* sp. and (**G**) *Pithecia irrorata*. The differences between the observation periods were calculated using analysis of variance with the Sidak test for paired multiple comparison. Data were compared between pre- and post-vaccination by ANOVA followed by Sidak’s multiple pairwise comparison test.

Additionally, considering individual observations, no adverse effects were observed, such as prostration, signs of pain, increased water consumption (which could occur in case of fever), decreased food consumption, or manipulation of the vaccination site due to discomfort from the injection (Figure 2).

### 3.2. Clinical and Health Evaluation

In addition to the behavioral analysis as a demonstration of adverse effects, physical exams and evaluations of blood count and biochemical profile were conducted. Each animal was assessed individually, with the information obtained in −15 DPV used as a control for comparisons after vaccination. Only one brown howler monkey (600ED23) had clinical signs during the pre-vaccination period, including diarrhea, apathy, neutrophilic leukocytosis, and emaciation. This animal was treated with an antimicrobial (metronidazole), with significant improvement by the time of vaccination. Other diagnoses included chronic vaginitis (00064D07D0) and tubo-ovarian cyst (F59F), which did not pose any risk of deleterious effects for immunization, so they were not considered contraindications for vaccination.

After vaccination, none of the vaccinated animals had statistically significant differences in the hematological and biochemical parameters. Sixty days after immunization, one *S. imperator* (00064CF8DA), two *L. cana* (231264 and 224298), and one *A. guariba clamitans* (232191) had lost between 11% and 21% of their initial weight but showed no other clinical changes (Table A1).

### 3.3. Post-Vaccination RNAemia

Immunization with the 17DD YFV strain resulted in detectable RNAemia at 4 DPV in 9 of the 24 animals (37.5%), including 3 out of 5 *S. xanthosternos* (00064D07D0, 901293 and F59F), 1 (the only) *P. irrorata* (00064D1804) and 5 (5/7) *A. guariba clamitans* (203689, 600ED23, 232191, 64D1D3E and 26497437) (Table 2). At the other time points, there was no detectable viral RNA in blood samples. There were no significant differences in IgG or neutralizing antibody levels or frequency of serologic positivity between animals with or without RNAemia at all time points. Furthermore, there was no correlation between Ct values of animals with RNAemia and OD values (indicator of IgG levels) or neutralizing antibody titers.

### 3.4. Post-Vaccination Immunogenicity

**Figure 3:**
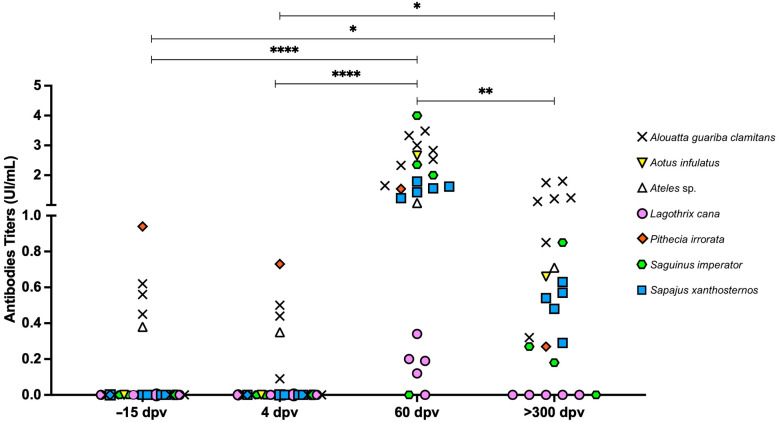
IgG levels in neotropical primates vaccinated with the attenuated yellow fever virus 17DD. Individual serum samples obtained prior to vaccination (−15 days post-vaccination [DPV]), at 4, 60, and 300 DPV were tested for total anti-yellow fever virus IgG by ELISA, with values >0 IU/mL considered positive. Differences between time points compared using the Friedman test (**** *p* < 0.0001; ** *p* < 0.01; * *p* < 0.05).

All vaccinated animals developed anti-YFV IgG at 60 DPV (Figure 3), except for one *L. cana* and one *S. imperator* (2643996 and 82644296, respectively), which did not seroconvert at any of the post-vaccination time points. The *P. irrorata*, four *A. guariba clamitans*, and an *Ateles* sp. had detectable IgG at −15 DPV and/or 4 DPV; however, the titers were higher at 60 and 300 DPV (Friedman, *p* < 0.0001 and *p* < 0.05, respectively).

**Figure 4:**
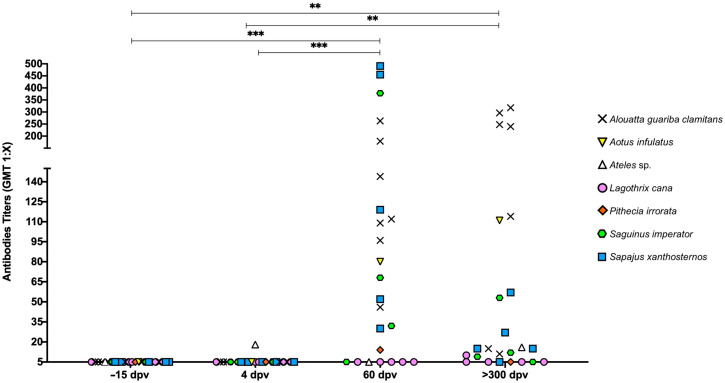
Neutralizing antibodies detected in neotropical primates vaccinated with the attenuated yellow fever virus 17DD. Individual serum samples obtained prior to vaccination (−15 days post-vaccination [DPV]), at 4, 60, and 300 DPV were tested for neutralizing activity with individual serum dilutions associated with a 50% reduction in viral infection on control plates. Differences between time points compared using the Friedman test (*** *p* < 0.001; ** *p* < 0.01).

There were species-specific differences on the vaccine-elicited neutralizing antibodies (Figure 4). All *L. cana* and one *S. imperator* (82644296) did not have titers at any post-vaccination time point, while the *Ateles* sp. had titers at 4 DPV, but was negative at 60 DPV. At 60 DPV, all other animals belonging to the following species: *S. imperator*, *S. xanthosternos*, *A. guariba clamitans*, *P. irrorata*, and *A. infulatus* developed neutralizing antibody titers. The pattern of serological response differed among species. Individuals of woolly and *Ateles* sp. and one *S. imperator* did not show neutralizing antibodies at 60 DPV, while at 300 DPV one *L. cana* produced neutralizing antibodies (1:10). However, there was no persistence of titers in one individual of *S. xanthosternos* (901293) and in the *P. irrorata*, as well as the other *L. cana* individuals.

## 4. Discussion

In this study, various species of neotropical primates were immunized, which is relevant since there are no previous reports of vaccination with the attenuated 17DD vaccine for *Saguinus imperator*, *Lagothrix cana*, *Sapajus xanthosternos*, *Pithecia irrorata*, *Aotus infulatus* and *Ateles* sp. Therefore, this study demonstrated the safety and efficacy of vaccination in many neotropical primate species and confirmed the safety of vaccination in *Alouatta* sp., which has been addressed in a previous study [10].

Assessment of vaccination-elicited IgG and neutralizing antibody responses demonstrate differences in the pattern of responses among different species of neotropical primates. One *S. imperator* did not have seroconversion at any time point, suggesting a possible failure in inoculation in that particular case, since the other individuals of the same species exhibited high levels of both IgG production and neutralizing antibodies. Importantly, the antibody levels persisted at 300 DPV. All *S. xanthosternos*, *A. guariba clamitans*, and the *A. infulatus* had seroconversion and persistent immunity as demonstrated by production of IgG and neutralizing antibodies. Evaluation of neutralizing antibody response in rhesus monkeys inoculated with graded doses of the 17DD vaccine induced a high degree of immunogenicity [20]. Similarly, immunization of *Alouatta* sp. with the attenuated human vaccine triggered the production of neutralizing anti-YFV antibodies [10], as previously shown by studies with the 17DD strain in rhesus monkeys [21], similar to responses observed in human patients [22].

Similar to what was observed in one *S. imperator*, one *L. cana* did not seroconvert but, unlike the *S. imperator*, the other four *L. cana* included in this study developed anti-YFV IgG antibodies at 60 DPV but failed to generate neutralizing antibodies. These results suggest that *L. cana* is not quite responsive to vaccination, as the neutralizing antibody response is what effectively prevents against natural exposure to YFV [23]. A previous study, in which *L. cana* were infected with the Asibi strain of YFV by direct inoculation or mosquito transmission, demonstrated that most of the animals died, although no lesions attributable to YF were observed, so it was concluded that the deaths were not caused by YFV [24]. With the neurotropic YFV and intracerebral inoculation, the monkeys developed severe encephalitis and died, but no visceral lesions were observed. A small number of woolly monkeys were inoculated with the YFV Asibi strain in Villavicencio, Colombia, with results similar to those of Davis (1930) [24]. There was no mortality directly attributable to the virus, but the monkeys showed moderate levels of viremia. The performance of serum in the mouse protection test, however, was similar to that for other primates, providing immunity against the virus in rodents [25]. Furthermore, it is evident that this species has varying susceptibility to the disease, with results differing from those observed in *A. guariba clamitans* and other species of neotropical primates.

The *Ateles* sp. had a different profile of immune response, as it developed neutralizing antibodies at 4 DPV (1:18), but became negative at 60 DPV and had a titer of 1:16 at 300 DPV. Similarly, the IgG response, which already indicated titers at −15 DPV (0.38 IU/mL) and 4 DPV (0.35 IU/mL), reached 1.07 IU/mL at 60 DPV and 0.71 IU/mL at 300 DPV. A previous report of infection in *Ateles paniscus* by inoculation and mosquito bite by *Aedes aegypti* identified the species as sensitive to the virus but, in two individuals, the expected necropsy findings in other neotropical primates were not observed, with no hepatic necrosis but severe renal damage, while other animals developed a febrile response and YFV levels in liver emulsion. The study concluded that the species is highly susceptible to the virus [25]. This information suggests variation in infection by YF virus in the genus, possibly representing a difference in immune response in this species, as the production of antibodies was transient in the context of our study.

The *P. irrorata* also had IgG titers at −15 DPV (0.94 IU/mL) and at 4 DPV (0.73 IU/mL), reaching 1.54 IU/mL at 60 DPV, and 0.27 IU/mL at 300 DPV. In this animal, there were detectable neutralizing antibodies at 60 DPV (dilution 1:14), indicating seroconversion. However, these antibodies did not persist at 300 DPV. This animal also had viremia at 4 DPV. In this species, there are no previous studies indicating the profile of infection by YFV, clinical signs or lesions observed. This is the first study to demonstrate the serological response profile after immunization.

Viremia after vaccination has been previously reported in howler monkeys [10] and rhesus monkeys [9], showing low and transient levels between the 4th and 7th days and 6 days post-immunization, respectively, which is consistent with our findings of viremia at 4 DPV, especially considering that this is a live attenuated virus. Given the variation in results between animals of the same species, it is suggested that, in addition to species characteristics, there is individual variation in the innate immune response. These results were also previously described in humans vaccinated for the first time with the 17DD strain, with RNAemia detected by RT PCR, from the second to the sixth day after vaccination [26].

It was not expected for the animals to have positive serology before vaccination, as occurred in some primates in this study. However, the test used may have cross-reactivity with other Ortho-flaviviruses, including dengue virus [27], which is highly prevalent in the area of this study. It is also important to consider that these non-human primates are housed in outdoor enclosures, so they are exposed to mosquito bites. The possibility that this was due to prior contact with the YFV is less likely, given that the common course of the disease is acute and highly lethal in NHPs, especially in *Alouatta* sp. [6], and there are no previous cases of YFV diagnosed in this zoo.

Considering the expansion of the geographic distribution of YF in Brazil and its effects on NHP populations, efforts to protect these species are extremely important. However, vaccinating primates is a topic of discussion, due to their role as sentinels favoring early detection of YFV circulation, which allows prompt control measures, such as vaccination of the human population in affected areas. Nonetheless, it is important to consider vaccinating these animals as a contingency measure to reduce viral circulation near areas with high human populations, preventing these animals from acting as virus reservoirs and sources of infection for mosquitoes, as well as protecting and preserving endangered species, which may be even more relevant than their role as sentinels. The proposed immunization of NHPs in monitored areas, such as zoos, research centers, parks, and forest reserves near urban areas, could effectively contribute to control efforts. This knowledge can support further studies with other susceptible NHP species and provide a possible solution for controlling epizootics and preventing the devastation of endangered species, offering a significant contribution to the prevention of epizootic outbreaks and the conservation of endangered species, particularly ex situ.

## 5. Conclusions

Our results demonstrated a safe, immunogenic and persistent protection provided by the attenuated 17DD YFV vaccine strain in *A. guariba clamitans*, *S. xanthosternos*, *S. imperator*, and *A. infulatus*. However, under the conditions of this study, the vaccine was not immunogenic for *L. cana*. In *P. irrorate,* seroconversion did not show long-term persistence, while the *Ateles* sp. had a transient immune response.

## Figures and Tables

**Table 1:** Neotropical primates that were immunized against yellow fever: species, identification, sex, age, and weight at the time of vaccination.

Microchip	Species	Common Name	Sex	Age	Weight (kg)
232180	*Saguinus imperator*	Emperor tamarin	Male	12 years	0.615
00064CF8DA	*Saguinus imperator*	Emperor tamarin	Male	14 years	0.455
82644296	*Saguinus imperator*	Emperor tamarin	Male	11 months	0.385
252601	*Saguinus imperator*	Emperor tamarin	Male	4 years	0.539
00064D07D0	*Sapajus xanthosternos*	Yellow-breasted capuchin	Female	16 years	2.78
901293	*Sapajus xanthosternos*	Yellow-breasted capuchin	Female	4 years	1.8
022197	*Sapajus xanthosternos*	Yellow-breasted capuchin	Male	7 years	3.9
F59F	*Sapajus xanthosternos*	Yellow-breasted capuchin	Female	13 years	3.18
493175	*Sapajus xanthosternos*	Yellow-breasted capuchin	Male	8 years	4.120
234113	*Lagothrix cana*	Woolly monkey	Female	10 years	5.76
231264	*Lagothrix cana*	Woolly monkey	Female	8 years	5.9
224298	*Lagothrix cana*	Woolly monkey	Female	7 years	6.065
2643996	*Lagothrix cana*	Woolly monkey	Female	2 years	3.75
203805	*Lagothrix cana*	Woolly monkey	Male	8 years	8.48
00064D1804	*Pithecia irrorata*	Gray’s bald-faced saki	Female	12 years	2.79
22194	*Aotus infulatus*	Owl monkey	Female	14 years	0.905
203689	*Alouatta guariba clamitans*	Brown howler monkey	Female	9 years	3.525
023759	*Alouatta guariba clamitans*	Brown howler monkey	Female	8 years	5.255
600ED23	*Alouatta guariba clamitans*	Brown howler monkey	Female	18 years	3.295
023756	*Alouatta guariba clamitans*	Brown howler monkey	Female	7 years	4.18
232191	*Alouatta guariba clamitans*	Brown howler monkey	Female	3 years	3.715
64D1D3E	*Alouatta guariba clamitans*	Brown howler monkey	Male	7 years	7.765
2649743	*Alouatta guariba clamitans*	Brown howler monkey	Female	9 months	1.215
204802	*Ateles* sp.	Spider monkey	Male	10 years	8.305

**Table 2:** Neotropical primates immunized against yellow fever with 4 DPV samples with quantification cycles (Ct) below 37, therefore considered a positive RNAemia.

Species	Microchip	Ct Value
*Sapajus xanthosternos*	00064D07D0	36
*Sapajus xanthosternos*	901293	32
*Sapajus xanthosternos*	F59F	35
*Pithecia irrorata*	00064D1804	29
*Alouatta guariba clamitans*	203689	28.1
*Alouatta guariba clamitans*	600ED23	29.36
*Alouatta guariba clamitans*	232191	33.59
*Alouatta guariba clamitans*	64D1D3E	29.85
*Alouatta guariba clamitans*	26497437	36.97

## Data Availability

All relevant data are included in this article and Appendix A.

## Abbreviations

The following abbreviations are used in this manuscript:

YF	Yellow Fever
YFV	Yellow Fever Virus
NHP	Non-human Primates
CITES	Convention on International Trade in Endangered Species
ICMBio	Instituto Chico Mendes de Conservação da Biodiversidade
DPV	Days Post-vaccination
RT–qPCR	Reverse Transcription followed by Polymerase Chain Reaction
SC	Subcutaneously
PRNT	Plaque Reduction Neutralization Test
PFU	Plaque Forming Units
CTL	Cellular Technology Limited
ELISA	Enzyme Linked Immunosorbent Assay
SBD	Blocking/Diluent Solution
BSA	Bovine Serum Albumin

## Appendix A

**Table A1:** Weight of the animals during the screening, 15 days before vaccination (−15 DPV), and at 4, 60, and 300 days post-vaccination (DPV).

Microchip	Species	−15 DPV	0 DPV	4 DPV	60 DPV	300 DPV
232180	*Saguinus imperator*	0.540	0.615	0.550	0.615	0.655
00064CF8DA	*Saguinus imperator*	0.480	0.455	0.455	0.395	0.500
82644296	*Saguinus imperator*	0.385	0.385	0.385	0.395	0.400
252601	*Saguinus imperator*	0.590	0.539	0.535	0.530	0.520
00064D07D0	*Sapajus xanthosternos*	2.810	2.780	2.785	2.970	2.980
901293	*Sapajus xanthosternos*	1.905	1.800	1.790	1.860	1.975
022197	*Sapajus xanthosternos*	4.040	3.900	3.870	3.860	3.875
F59F	*Sapajus xanthosternos*	3.270	3.180	3.170	3.010	3.755
493175	*Sapajus xanthosternos*	4.205	4.120	4.125	4.185	4.165
234113	*Lagothrix cana*	6.000	5.760	5.765	5.760	5.700
231264	*Lagothrix cana*	6.120	5.900	5.860	5.460	5.680
224298	*Lagothrix cana*	6.320	6.065	6.060	5.540	5.675
2643996	*Lagothrix cana*	3.755	3.750	3.750	3.830	4.275
203805	*Lagothrix cana*	8.680	8.480	8.480	8.765	8.900
00064D1804	*Pithecia irrorata*	3.000	2.790	2.780	2.885	3.130
22194	*Aotus infulatus*	0.930	0.905	0.915	1.000	0.985
203689	*Alouatta guariba clamitans*	3.825	3.525	3.520	3.760	4.020
023759	*Alouatta guariba clamitans*	5.655	5.255	5.250	5.250	5.480
600ED23	*Alouatta guariba clamitans*	2.975	3.295	3.305	3.555	3.795
023756	*Alouatta guariba clamitans*	4.220	4.180	4.190	4.175	4.645
232191	*Alouatta guariba clamitans*	4.140	3.715	3.700	3.230	4.150
64D1D3E	*Alouatta guariba clamitans*	8.185	7.765	7.830	8.630	8.605
2649743	*Alouatta guariba clamitans*	1.200	1.215	1.265	1.600	2.595
204802	*Ateles* sp.	8.140	8.305	8.380	8.915	7.885
